# Human T-cell lymphotropic virus screening in France: Missed opportunities? A retrospective multicenter study of serological testing in hospital laboratories

**DOI:** 10.1016/j.ijregi.2024.100387

**Published:** 2024-06-13

**Authors:** Olivier Moquet, Ibrahima Faye, Nicolas Auffret, Benoit Garin, Ségolène Brichler, Raymond Césaire

**Affiliations:** 1 Centre de biologie du Nivernais, centre hospitalier de l'agglomération de Nevers, Nevers, France; 2 Plateforme de Biologie Hospitalo-Universitaire, secteur virologie, centre hospitalo-universitaire de Dijon, Dijon, France; 3 Laboratoire de Virologie, centre hospitalo-universitaire de la Guadeloupe, Pointe-à-Pitre, France; 4 Service de microbiologie clinique, hôpital universitaire Avicenne, Bobigny, France; 5 PCCEI, University of Montpellier, Inserm, EFS and University of Antilles, Pointe-à-Pitre, France

**Keywords:** Human T-lymphotropic virus 1, Breastfeeding, Antenatal screening, Sexually transmitted infection, Blood-borne infection

## Abstract

**Objectives:**

Human T-lymphotropic virus 1 infection is endemic in the French Antilles, French Guiana, and sub-Saharan Africa, the origin of many immigrants currently living in France. There are no national screening recommendations outside of the regulatory obligations concerning donations of blood, tissue, gametes, or milk to a lactarium. This study aimed to investigate the screening and diagnostic practices for this infection in France.

**Methods:**

Serological statistics for regulatory, antenatal, sexually transmitted infections (for CeGIDDs (Centre Gratuit d'Information, de Dépistage et de Diagnostic des Infections Sexuellement Transmissibles), which are public sexual health clinics), accidental exposure to blood screenings, and diagnosis since January 1, 2018 were collected from 23 hospital laboratories (two in the French Antilles, 21 in mainland France) associated with 55 hospitals and 22 maternity units.

**Results:**

A total absence of antenatal screening was reported by 75% of the laboratories associated with maternity units in mainland France. All the laboratories in mainland France reported an absence of screening in the accidental exposure to blood context, as did all the laboratories in mainland France associated with a CeGIDD in the context of sexually transmitted infection screening. Conversely, screening in accordance with the existing regulations was generally systematically carried out. The most frequently reported diagnostic contexts were hematology and neurology.

**Conclusions:**

This study reveals an underscreening of human T-lymphotropic virus 1 in the hospital laboratories of mainland France.

## Highlights

• Survey of serological testing for human T-cell lymphotropic virus in French hospital laboratories.
• No antenatal screening performed in 75% of laboratories in mainland France.
• No screening performed after accidental exposure to blood in mainland France.
• No screening in the context of sexually transmitted infections in mainland France.
• The development of national recommendations for screening would be useful.

## Introduction

Human T-cell lymphotropic virus type (HTLV) 1 (HTLV-1) was the first pathogenic human retrovirus to be discovered in 1980, 3 years before HIV [1]. Comparable its “famous distant cousin,” this virus principally infects clusters of differentiation 4^+^ T lymphocytes. HTLV-1 is transmitted from cell to cell, and viral particles are not detected in cell-free plasma. The virus can be transmitted from mother to child (estimated mean risk of 15%, almost exclusively through breastfeeding, with the risk increasing further if breastfeeding is prolonged), unprotected sex (mainly from men to women), and contact with blood or tissues containing infected cells.

It has been estimated that at least 5-10 million people worldwide are infected with the virus. However, HTLV-1 prevalence data are based on studies covering only 1.5 billion people and, therefore, probably represents a considerable underestimate. Based on the available data, the virus is present on the five main continents (Europe, Asia, America, Africa, and Oceania) but with a highly heterogeneous geographic and ethnic distribution, with endemic regions often situated next to areas documented to be free from contamination. The reasons for this pattern are unknown but may include a founder effect, at least in certain populations, and significant subsequent persistent transmission [2].

The main known areas in which this virus is endemic (prevalence ≥1% in the adult population) are the Caribbean, South America, sub-Saharan Africa, southwest Japan, and parts of the Middle East and Oceania. The French overseas territories located in the Americas (Guadeloupe, Martinique, French Guiana) are recognized endemic areas [2].

HTLV-1 is known principally as the cause of two severe diseases. The first is adult T-cell leukemia/lymphoma (ATLL) associated with HTLV, a malignant proliferation of CD4+ T lymphocytes. The acute leukemic form is the most aggressive hemopathy known, and the median survival for the lymphoma and leukemic forms is <1 year, a dismal prognosis due to its high proliferation, immunosuppression, and chemoresistance [3]. The second well-known HTLV-related disease is tropical spastic paraparesis/HTLV-associated myelopathy (TSP/HAM), a chronic progressive neuromyelopathy, affecting principally the lower dorsal spinal cord. After 10 years of progression, half the patients are wheelchair-bound [4].

ATLL develops in approximately 3-7% of infected patients and TSP/HAM in approximately 0.5-3% after a period of clinical latency lasting several years for TSP/HAM and decades for ATLL [5]. ATLL develops almost exclusively in patients infected during breastfeeding in infancy, with the overall risk in this population estimated at up to 25% over the course of a lifetime [6].

The development of other inflammatory syndromes (uveitis, alveolitis, myositis, dermatitis, etc.) or infectious syndromes linked to virus-induced immunosuppression (e.g. strongyloides hyperinfection syndrome) has also been shown to be associated with the virus [5], again, in a minority of patients. Thus, the vast majority of infected patients are clinically asymptomatic and most will remain so. However, the spectrum of conditions associated with the virus probably remains largely unknown. In a recent meta-analysis, individuals infected with HTLV-1 have a 57% higher global risk of early death that cannot be explained by current knowledge [7]. HTLV-1 infection is not silent and HTLV-1–mediated inflammation may have a broader impact than generally recognized. There are clinical research gaps on HTLV-1 and cardiovascular or cerebrovascular conditions. HTLV-1–associated pulmonary disease may be more severe than initially concluded in early case series [8]. Concerns has been raised on the potential impact of HTLV-1 in chronic kidney disease and diabetes [9]. HTLV-1 infection has been associated with dysthyroidism in French Guiana [10].

In the French Caribbean territories, HTLV-1 antibodies screening in blood donors and prevention of mother-to-child transmission have been implemented since 1989. In mainland France, HTLV screening is mandatory in blood donors since mid-July 1991 and has been limited to first-time blood donors since 2017 [11]. In France, systematic screening for HTLV-1 is also mandatory for all donations of milk to a lactarium [12], organs, tissues, [13], and gametes [14]. Outside of the French Antilles and French Guiana, representative national data on the overall prevalence of HTLV-1 infection in France are scarce. The only national data regularly collected are those from the screening of blood donations. The total national prevalence of HTLV-1 infection has been estimated at about 0.005% in the population of new blood donors, in which individuals from endemic areas are very strongly underrepresented and, systematically, subjected to upstream selection based on the risk of transmission of infectious agents [15]. Nevertheless, this estimated prevalence among new donors is the second highest in Europe after Romania, the only European territory in which HTLV-1 is considered to be endemic [2,16]. Other European countries mainly affected by HTLV-1 infection based on migration flows from endemic regions are the United Kingdom (UK) and Spain [17]. A recent update of the estimated prevalence of HTLV-1 in the UK showed an increase from 14,900 in 1991 to 36,300 in 2021, supported by an increase annual incidence of ATLL [17].

Nearly half the infected donors screened in mainland France come from endemic regions (mostly the French Antilles and sub-Saharan Africa) and one-third report one or more sexual partners from an endemic area as the only risk factor [16]. A study performed in 2005 reported a prevalence of HTLV-1 infection of 0.115% in a sample of 10,398 pregnant women from the Parisian region [18], a rate lower than that for HIV infection but of a similar order of magnitude.

However, outside of the contexts covered by regulatory screening, there are no national screening recommendations, either untargeted or targeting these populations at risk, particularly, in the antenatal context. It has been estimated that more than 1.25 million immigrants and 890,000 descendants of immigrants of sub-Saharan origin live in mainland France, with a trend toward an increase in this population since the start of the century in terms of absolute numbers and as a proportion of the total population [19]. It has also been estimated that more than one-third of individuals aged between 15 and 64 years born in the French Antilles or French Guiana live in France but outside their region of origin [20]. The aim of this survey was to evaluate the screening and diagnostic practices for HTLV-1 infection in different regulatory and/or risk contexts in France.

## Materials and methods

Hospital laboratories were asked to participate in a retrospective survey of their serological screening and diagnostic activity for HTLV-1 infection (whether performed on-site or subcontracted) since January 1, 2018. Members of the College of Bacteriology/Virology/Hygiene of general hospitals (colBVH), the French Society of Microbiology (SFM), and the medical microbiology network (R2M) were asked to participate via their respective electronic mailing lists. The survey took place from September 7 to November 20, 2023.

For each participating laboratory, data were collected by the local investigator querying the laboratory information system. The overall number of biological tests performed and the number performed by context were obtained by a statistical informatic query based on the unique code associated with the test in the laboratory information system setting or in the French nomenclature of medical biology acts.

The context of the prescription retained by the local investigator for each request was defined based on the medical specialty of the prescriber and, possibly, other examinations and/or the indication or clinical information mentioned in the medical biology file. The diagnostic contexts investigated were hematology, neurology, dermatology, ophthalmology, or other contexts known to the laboratory.

The screening contexts investigated were antenatal screening, screening for milk donation to the lactarium, screening for graft/tissue donations, screening after accidental exposure to blood (AEB), and screening carried out at public sexual health clinics (CeGIDD - Centre Gratuit d'Information, de Dépistage et de Diagnostic des Infections Sexuellement Transmissibles). Except for screening for CeGIDDs, laboratories were also asked whether, for each clinical context, a local protocol mentioned when to screen for HTLV. Laboratories were also asked to specify the proportion of patients for which HTLV screening was performed, in a semi-quantitative manner, by screening context (<0.1%, from 0.1 to 1%, from 1 to 10%, from 10 to 50%, >50%).

The data from each participating laboratory were entered into the database by the local investigator via an online form published on the jotform platform (www.jotform.com). The results for screening activity are summarized in Table 1 and those for diagnostic activity are summarized in Table 2.

**Table 1:** Summary of the results for screening activity.

Number of laboratories	2	4	1	10	2	2	1	1
Localization	FWI	MF	MF	MF	MF	MF	MF	MF
Number of affiliated hospitals	6	5	1	29	7	4	1	2
Medicine, Surgery an Obstetrics beds	1400	3286	700	8842	2325	1300	250	380
Associated with a maternity unit	Y	Y	Y	Y	Y	Y	Y	N
Deliveries 2022	4600	10391	4900	15767	9456	5850	750	
Associated with a lactarium	Y	Y	Y	N	N	N	N	N
Total tests per year	6698	2220	523	1158	1756	407	6	59
Antenatal screening	Y	Y	Y	Y	Y	Y	Y	N
HTLV antenatal screening	Y	N	N	N	Y	Y	Y	
HTLV antenatal screenings per year	3262				186	2	4,1	
Proportion of patients screened for HTLV	≥50%				1 to 10%	< to 0.1%	0,1 to 1%	
Protocol with HTLV	Y (1/2) N (1/2)	N	N	N	Y (1/2) N (1/2)	N	N	N
Screening for milk donation	Y	Y	Y	Y (1/10) N (6/10) NA (1/10)	Y (1/2) N (1/2)	N	N	N
HTLV screening for milk donation	Y	Y	Y	Y (1/1)	Y (1/1)			
HTLV screenings for milk donation per year	982	800	NA	<1 (1/1)	NA (1/1)			
Proportion of patients screened for HTLV	≥50%	≥50% (3/4) NA (1/4)	≥50%	≥50% (1/1)	≥50% (1/1)			
Protocol with HTLV	Y	Y	NA	Y (2/10) N (8/10)	Y (1/2) N (1/2)	N	N	N
Screening for “graft”	Y	Y (1/4) N (1/4) NA (2/4)	NA	Y (5/10) N (4/10) NA (1/10)	N	Y (1/2) N (1/2)	N	Y
HTLV screening for “graft”	Y	Y (1/1)		Y (4/5) NA (1/5)		Y (1/1)		NA
HTLV screenings for “graft” per year	217	99 (1/1)		234 (4/4)		37 (1/1)		
Proportion of patients screened for HTLV	≥50%	≥50% (1/1)		≥50% (4/4)		NA		
Protocol with HTLV	Y	Y (3/4) N (1/4)	N	Y (7/10) N (3/10)	N	Y (1/2) N (1/2)	N	N
HTLV screening after AEB	Y	N	N	N	N	N	N	N
HTLV screening after AEB per year	103							
Proportion of patients screened for HTLV	≥50%							
Protocol with HTLV	Y	N	N	N	N	N	N	N
Screenings for CeGIDD	N	Y	N	Y (4/10) N (6/10)	Y	Y (1/2)N (1/2)	Y	N
HTLV screenings for CeGIDD		N		N (4/4)	N	N (1/1)	N	

AEB, accidental exposure to blood; FWI, French West Indies; HTLV, human T-lymphotropic virus; MF, mainland France; N, no; NA, not answered; Y, yes - a fraction indicates the proportion of laboratories concerned.

**Table 2:** Summary of the results for diagnostic activity.

Number of laboratories	1	3	3	4	2	1	1
Localization	FWI	MF	MF	MF	MF	MF	MF
Number of affiliated hospitals	1	5	10	10	6	2	4
Medicine, Surgery, and Obstetrics beds	600	2507	3212	1850	1200	1079	1624
Total tests per year	4067	2005	242	258	83	341	548
Hematology diagnosis context	Y	Y	Y	Y	NA	NA	Y
tests per year	102	509 (2/3) NA (1/3)	7 (2/3) NA (1/3)	25			65
Neurology diagnosis context	Y	Y	Y	N (1/4) NA (3/4)	Y	Y	Y
Tests per year	424	47 (2/3) NA (1/3)	14 (2/3) NA (1/3)		4	91	17
Dermatology diagnosis context	Y	Y	N	N (1/4) NA (3/4)	NA	N	Y
tests per year	11	12 (2/3) NA (1/3)					4
Ophthalmology diagnosis context	NA	NA (2/3) N (1/3)	N	N (2/4) NA (2/4)	Y	N	Y
Tests per year					2		15
Other known diagnostic context	NA	NA	N	NA	Y	N	NA
Tests per year					7		

FWI, French West Indies; MF, mainland France; N, no; NA, not answered; Y, yes - a fraction indicates the proportion of laboratories concerned.

## Results

### Participating laboratories

**Figure 1:**
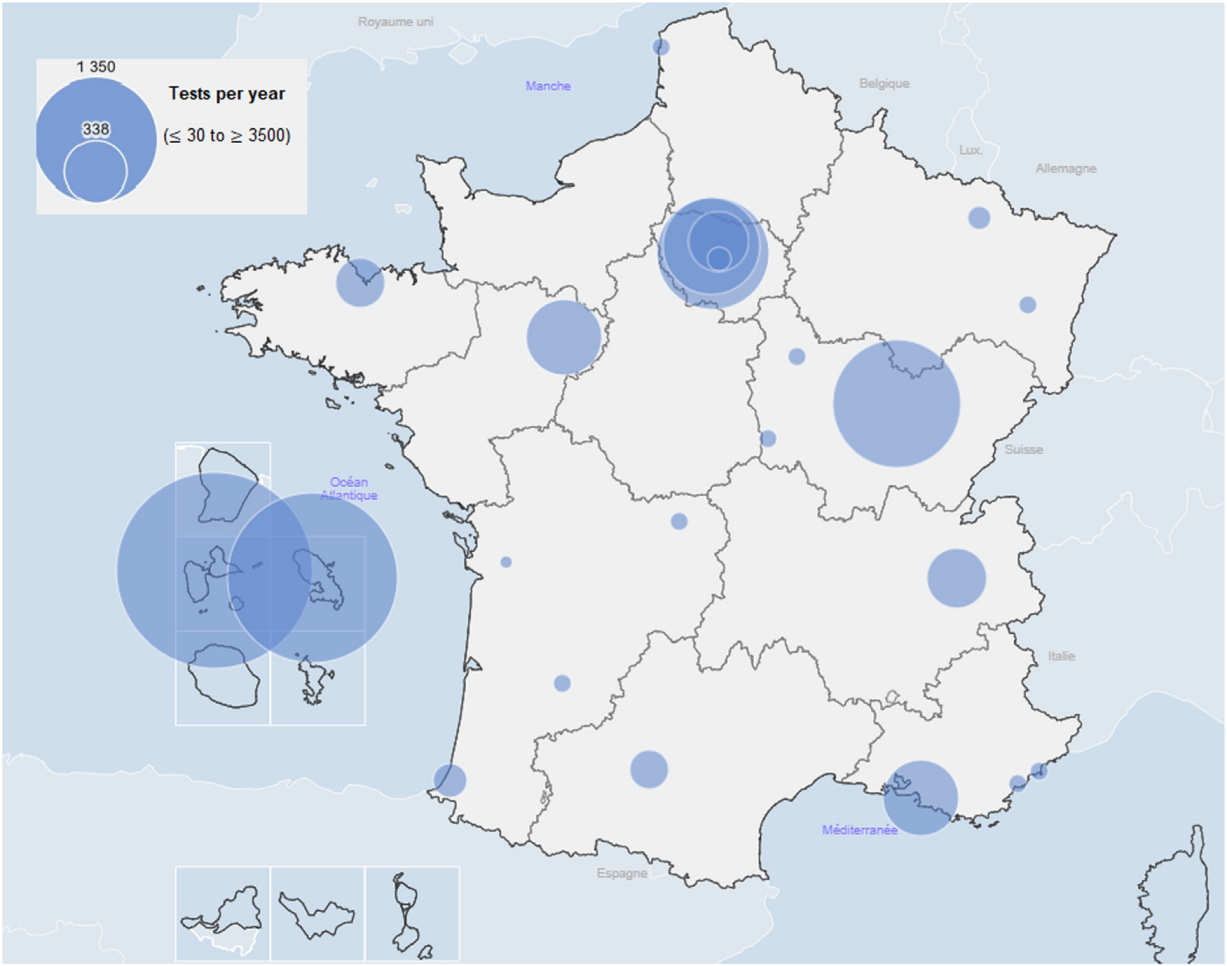
Geographic location and relative overall activities of the participating laboratories (≤30-≥3500 tests per year). This map was created using the Geodes website (https://geodes.santepubliquefrance.fr).

In total, 24 hospital laboratories responded to the survey. A total of 23 indicated that they could exhaustively retrieve the HTLV serology requests sent to them since January 1, 2018 (or since another more recent date for four), resulting in 23 exploitable responses. Two of the laboratories were located in the French Antilles (one in Guadeloupe and one in Martinique) and 21 were located in mainland France (Figure 1). A total of 10 laboratories carried out serological screening on-site and 13 subcontracted this work.

The 23 laboratories carried out medical biology tests for 55 hospitals, with a total capacity of 18,483 short-stay hospitalization beds in Medicine, Surgery, and Obstetrics. A total of 22 laboratories indicated the presence of a maternity unit within their hospital or hospital group. These 22 maternity units managed 51,714 deliveries in 2022, accounting for almost 7.2% of the 722,000 births estimated in France [21] (without taking multiple births into account). Seven laboratories indicated the presence of a lactarium within their hospital or hospital group.

The 23 laboratories handled a mean of 12,827 serology examinations per year (from 2 to 4067, depending on the laboratory). The two laboratories of the French Antilles alone performed a mean of 6,698 serology examinations per year (i.e. 52.2% of the total volume).

### Antenatal screening

A total of 22 laboratories (corresponding to the 22 laboratories indicating the presence of a maternity ward within their hospital group) indicated that they performed antenatal serological screenings (initial and/or follow-up). A total of 15 of these laboratories indicated that they did not cover HTLV screening as part of these assessments. This number corresponds to 68.2% of the laboratories performing examinations for maternity wards (75% of the laboratories in mainland France performing examinations for maternity wards) and 60.1% of the total deliveries (31,058 of 51,714) reported for these maternity wards in 2022 (65.9% of the total deliveries in mainland France).

Among the seven laboratories that carried out HTLV screening in this context, the two laboratories in the French Antilles indicated that such screening was performed for >50% of pregnant women. Such screening was performed sporadically for <0.1% of pregnant women at two of the five laboratories performing such screening in mainland France for 0.1-1% of pregnant women at another laboratory and for 1-10% of pregnant women at the two remaining laboratories.

Only two laboratories reported the existence of a local protocol indicating when to offer HTLV screening in an antenatal context: one in the French Antilles (mention of the systematic offer of screening to all future mothers) and one in mainland France, which reported performing HTLV screening for 0.1-1% of patients (screening systematically offered to all future mothers who planned to breastfeed).

### Screening of donations of milk to a lactarium

Nine laboratories indicated that they had carried out HTLV screening for milk donations to a lactarium (including the seven laboratories indicating the presence of a lactarium within their hospital group). Eight laboratories indicated that HTLV screening was carried out for >50% of milk donations and the remaining laboratory was unable to respond on this point.

Eight of these nine laboratories and one other laboratory indicated the existence of a local protocol specifying the HTLV screening procedures for milk donations (systematically for all donations).

Of note, six of the seven laboratories in mainland France (85.7%) that handled HTLV screening for milk donations (and six of the seven laboratories in mainland France [85.7%] reporting the existence of a local screening protocol for milk donations) reported that they had not performed screening in the antenatal context.

### Screening for graft/tissuedonation

A total of 10 laboratories indicated that having performed serological assessments for grafts/donated tissues. Eight of these laboratories indicated that they performed serological tests for HTLV in the framework of these assessments (the other two were unable to respond on this point). Nine of these 10 laboratories and four others indicated that they had a local protocol describing HTLV screening methods in this context (systematic). Seven of the eight laboratories that reported performing HTLV screening in this context indicated that HTLV screening was performed in >50% of assessments in this context (one laboratory was unable to respond on this point).

### Screening after accidental exposure to blood

Only two laboratories (the two laboratories in the French Antilles) indicated that they performed HTLV screening in the context of AEB. These two laboratories were also the only laboratories to indicate that HTLV was mentioned in their local AEB protocol (systematic screening of the source patient).

### Screening for CeGIDD

A total of 12 laboratories in mainland France reported carrying out serological assessments for one or more CeGIDDs. These 12 laboratories indicated that they had never performed serological tests for HTLV in this context.

### Diagnostic requests by clinical context

Eight laboratories were unable to provide information on the indication for serological testing requests in a diagnostic context.

A total of 15 laboratories reported one or more known indications: 12 reported performing serological tests in a hematologic context (three were unable to respond on this point for this indication), 11 in a neurological context (one indicated not performing the assessment and three were unable able to respond on this point for this indication), five in a dermatological context (five indicated not performing the assessment and five were unable to respond on this point for this indication), and three reported performing such tests in an ophthalmological context (seven indicated that they did not perform the assessment and five were unable to respond on this point for this indication). Two of the 16 laboratories also reported serological testing in another known diagnostic context (the indication was specified by one laboratory: request for systematic serological testing for any patient originating from an endemic area and hospitalized in the infectious diseases unit since the arrival of a practitioner who had worked in French Guiana).

### Discovery of an infection

Over the period of the study, seven laboratories (five in mainland France and two in the French Antilles) indicated that they had discovered HTLV infections, 11 (all in mainland France) reported that they had not detected any cases of infection, and five were unable to respond on this point. Among the seven laboratories reporting the detection of an infection, four in mainland France were able to specify a total of 36 diagnoses. Three of these laboratories reported the clinical contexts for all or some of the findings: three detections of infection occurred in a hematologic context, one in a neurological context, and one in the context of assisted human reproduction procedure.

## Discussion

In contrast to the situation in the French Antilles, we found that HTLV-1 screening activities occurred at a low level or not at all in mainland French laboratories, as part of the clinical activities of the corresponding hospital group or their clients (CeGiDD), with the exception of contexts in which screening is mandatory, in which, on the contrary, screening was subject to defined protocols and, above all, was carried out systematically.

This finding raises several questions.

However, the risk of contamination in certain regulatory contexts does not necessarily appear to be higher for the same route of transmission, particularly, for the general population, with a low apparent prevalence, and for the targeted population at risk. For example, inactivation of the virus by pasteurization [22] and shorter periods of exposure for milk donated to the lactarium result in a lower risk than breastfeeding with untreated milk.

Similarly, a significant reduction of the contagiousness of donated blood products through leukoreduction [23] results in a lower risk than exposure to blood in an accidental or drug-related context.

However, statistics on the origins and fluxes of the French population and the epidemiological data available for regions of known HTLV endemicity suggest that infection rates may be high in certain risk groups in mainland France.

The observed low rates of screening in mainland France can be explained by several factors that may be correlated with each other: few or no representative prevalence data available, (thus) an absence of recommendations from scientific societies and reference health agencies, and (thus) a lack of knowledge about the virus and/or its routes of transmission among some of the health actors concerned [24].

This study is subject to several real or potential biases that might affect its representativeness, including the following:

- The proportion of at-risk patients managed by these laboratories in mainland France is unknown.
- Patients may have been screened for the same indications in private laboratories (monitoring of pregnancies by private midwives, sexually transmitted infection screening prescribed by the attending physician, etc.).
- Representation bias (the investigators agreeing to participate may be those who had already noted a problem of underscreening in their laboratory).

However, these results indicate that there is underscreening, at least in mainland France, in situations in which there is a risk of transmission but for which there are no recommendations or regulatory obligations for testing. Better knowledge of the epidemiology of the virus in France based on truly representative data (which could be obtained through compulsory notification of findings of seropositivity, for example) and the development and dissemination of national recommendations for HTLV screening would be useful.

The World Health Organization has recently recognized HTLV-1 as a neglected infection of global concern. The 2021 World Health Organization Technical Report on HTLV-1 called for the strengthening of global public health measures against its spread [25]. HTLV-1 antenatal screening has been shown to be cost-effective in Brazil [26] and has been recently widespread at the national level. Targeted antenatal screening is under discussion in the UK (Graham Taylor, personal communication). Along with targeted screening of pregnant women, blood donors, and people who attended clinics for sexually transmitted infection, once-in-life testing has been suggested for HTLV-1, as recommended for HIV and hepatitis B and C [27]. Concerns have been raised about the need to provide information about transmission-preventive measures for people living with HTLV-1/2 [28]. Studies are also required on HTLV-1 transmission and HIV pre-exposure prophylaxis. Longitudinal clinical/biological follow-up of people living with HTLV-1 is now required based on recent studies that have shown the possibility of identifying people at risk of HAM/TSP [29] or patients at a pre-ATLL stage [30].

## Conclusion

This study reports low levels of screening activity or even the absence of screening for HTLV-1 infection in mainland France in the antenatal context and in screening for sexually transmitted diseases and after AEB in a network of hospital laboratories. Improvements in our knowledge of the national epidemiology of the virus and the development of national recommendations for its screening would be desirable.

## Declarations of competing interest

The authors have no competing interests to declare.
